# RNA Expression of DNA Damage Response Genes in Muscle-Invasive Bladder Cancer: Influence on Outcome and Response to Adjuvant Cisplatin-Based Chemotherapy

**DOI:** 10.3390/ijms22084188

**Published:** 2021-04-18

**Authors:** Jonas Herrmann, Helena Schmidt, Katja Nitschke, Cleo-Aron Weis, Philipp Nuhn, Jost von Hardenberg, Maurice Stephan Michel, Philipp Erben, Thomas Stefan Worst

**Affiliations:** 1 Department of Urology, University Medical Centre Mannheim, 68167 Mannheim, Germany; uro-direktion@umm.de (H.S.); Katja.nitschke@umm.de (K.N.); philipp.nuhn@umm.de (P.N.); Jost.vonhardenberg@umm.de (J.v.H.); Maurice-stephan.michel@umm.de (M.S.M.); Philipp.erben@medma.uni-heidelberg.de (P.E.); Thomas.worst@umm.de (T.S.W.); 2 Institute for Pathology, University Medical Centre Mannheim, 68167 Mannheim, Germany; Cleo-Aron.Weis@umm.de

**Keywords:** muscle-invasive bladder cancer, DNA-damage response, *BCL2*, *BRCA1*, *BRCA2*, *ERCC2*, *ERCC6*, *FOXM1*, *RAD50*, *RAD51*, *RAD52*, cisplatin, adjuvant chemotherapy

## Abstract

Background: Perioperative cisplatin-based chemotherapy (CBC) can improve the outcome of patients with muscle-invasive bladder cancer (MIBC), but it is still to be defined which patients benefit. Mutations in DNA damage response genes (DDRG) can predict the response to CBC. The value of DDRG expression as a marker of CBC treatment effect remains unclear. Material and methods: RNA expression of the nine key DDRG (*BCL2*, *BRCA1*, *BRCA2*, *ERCC2*, *ERCC6*, *FOXM1*, *RAD50*, *RAD51*, and *RAD52*) was assessed by qRT-PCR in a cohort of 61 MICB patients (median age 66 y, 48 males, 13 females) who underwent radical cystectomy in a tertiary care center. The results were validated in the The Cancer Genome Atlas (TCGA) cohort of MIBC (*n* = 383). Gene expression was correlated with disease-free survival (DFS) and overall survival (OS). Subgroup analyses were performed in patients who received adjuvant cisplatin-based chemotherapy (ACBC) (Mannheim *n* = 20 and TCGA *n* = 75). Results: Low expression of *RAD52* was associated with low DFS in both the Mannheim and the TCGA cohorts (Mannheim: *p* = 0.039; TCGA: *p* = 0.017). This was especially apparent in subgroups treated with ACBC (Mannheim: *p* = 0.0059; TCGA: *p* = 0.012). Several other genes showed an influence on DFS in the Mannheim cohort (*BRCA2*, *ERCC2*, *FOXM1*) where low expression was associated with poor DFS (*p* < 0.05 for all). This finding was not fully supported by the data in the TCGA cohort, where high expression of *FOXM1* and *BRCA2* correlated with poor DFS. Conclusion: Low expression of *RAD52* correlated with decreased DFS in the Mannheim and the TCGA cohort. This effect was especially pronounced in the subset of patients who received ACBC, making it a promising indicator for response to ACBC on the level of gene expression.

## 1. Introduction

Bladder cancer (BC) is the ninth most commonly diagnosed cancer worldwide [1]. Radical cystectomy (RC) with optional perioperative chemotherapy is the standard of care in muscle-invasive bladder cancer (MIBC) [2]. The five-year, disease-specific survival rate with RC alone is only 50–67% [3,4]. Perioperative cisplatin-based chemotherapy (CBC) may improve long-term survival and has been used since the 1980s [5]. However, only 30–40% of MIBC are responding to CBC [6]. Responders show improved survival rates [5,7,8]. Selection for perioperative CBC currently relies on clinicopathological features associated with a high risk of recurrence [2]. Concerns about overtreatment and the limited response rates cause poor implementation of perioperative CBC in clinical practice. Despite guideline recommendation and robust data [2,5], neoadjuvant CBC is administered only in around 30% of the applicable patients in the USA and in even less in many European countries [9].

Development of biomarkers to accurately identify responders could improve patient selection on the path to a more personalized approach to perioperative CBC.

Several targets have been investigated. It has been shown that different molecular subtypes of BC have shown differences in CBC response [10]. In addition, mutations in DNA damage response genes (DDRG) have been associated with increased sensitivity to CBC [11,12,13,14]. A correlation between DDRG and molecular subtypes has not been investigated.

A major problem in the clinical application of DDRG alterations as a marker of CBC response is the fact that mutations in the particular genes are seldom seen and profiling of the genes with relevance for DNA-damage response makes panel sequencing necessary. In addition, as gene expression is quantitatively obtainable in all samples, RNA expression could give information not only for those patients carrying a specific mutation. Furthermore, it can easily be assessed using standardized qRT-PCR assays.

Therefore, we aimed to evaluate the relevance of RNA expression of several DDRG in a MIBC cohort from a high-volume tertiary care center and The Cancer Genome Atlas (TCGA) MIBC dataset.

## 2. Results

### 2.1. Basic Characteristics of Patients

Patient characteristics of the Mannheim cohort are given in Table 1.

Patient characteristics of the TCGA cohort are given in Table 2.

### 2.2. Mannheim Cohort: All Patients

To investigate the impact of nine genes known to code for proteins relevant for DNA damage response on MIBC outcome we analyzed an unselected cohort of 61 patients who underwent radical cystectomy in the Department of Urology and Urosurgery of the University Medical Centre Mannheim. The expression of none of the genes differed between male and female, younger and older (≤70 years vs. >70 years), tumor stage T2 vs. tumor stage T3/4, and negative lymph nodes (N0) vs. positive lymph nodes (N+) at the time of cystectomy. Spearman correlation analyses revealed highly significant positive correlations among all combinations of genes, except for *FOXM1* and *BCL2*. Correlation coefficients (ρ) ranged from 0.4303 (*p* = 0.0032) for *BCL2* and *RAD51* to 0.8746 (*p* < 0.0001) for *BRCA1* and *BRCA2* (Supplementary Table S1).

**Figure 1:**
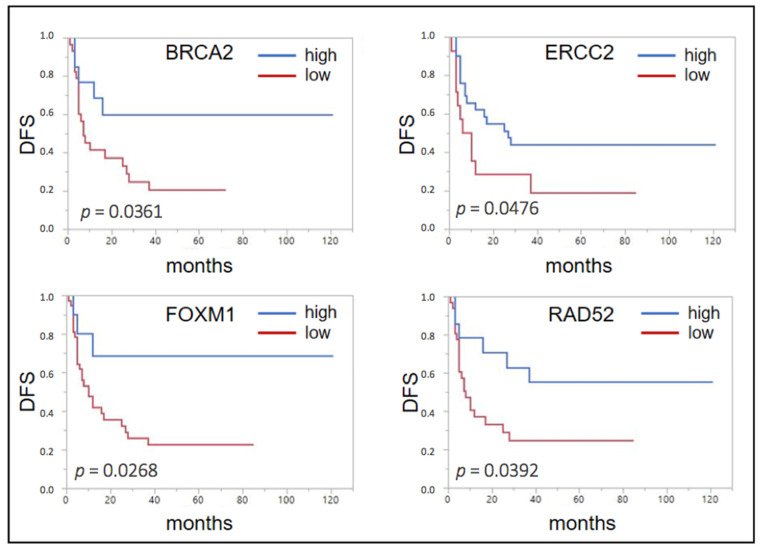
Forrest plots of the correlation between RNA expression and disease free survival (DFS) in the unselected Mannheim cohort.

After cutoff determination using the partition test, the expression of none of the nine selected genes showed a significant association with overall survival (OS). Yet, for disease-free survival (DFS) a significant association could be seen for a lowered expression of *BRCA2* (*p* = 0.0361, median DFS 7 months (mo) vs. not reached (n.r.)), *ERCC2* (*p* = 0.0476, 8 vs. 27 mo), *FOXM1* (*p* = 0.0268, 10 mo vs. n.r.), and *RAD52* (*p* = 0.0392, 8 mo vs. n.r.) (Figure 1).

### 2.3. Mannheim Cohort: Patients Treated with ACBC

**Figure 2:**
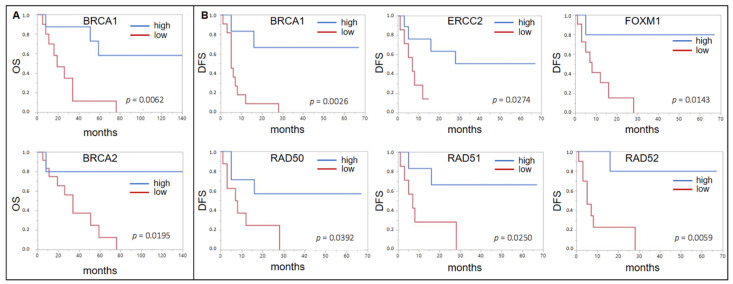
(**A**) Forrest plots of the correlation between RNA expression and overall survival (OS) in the Mannheim adjuvant cisplatin based chemotherapy (ACBC) cohort. (**B**) Forrest plots of the correlation between RNA expression and DFS in the Mannheim ACBC cohort.

Using the same cutoff determination method for the subcohort of 20 patients who received ACBC, a low expression of *BRCA1* (*p* = 0.0062, median OS 19 mo vs. n.r.) and *BRCA2* (*p* = 0.0195, 36 mo vs. n.r.) was associated with a shorter OS (Figure 2A). For *BRCA1* (*p* = 0.0026, median DFS 5 mo vs. n.r.), *ERCC2* (*p* = 0.0274, 7 mo vs. n.r.), *FOXM1* (*p* = 0.0143, 8 mo vs. n.r.), *RAD50* (*p* = 0.0392, 7.5 mo vs. n.r.), *RAD51* (*p* = 0.0250, 7 mo vs. n.r.), and *RAD52* (*p* = 0.0059, 5 mo vs. n.r.), this was also seen for DFS (Figure 2B). *BRCA2* almost reached significance (*p* = 0.0644, 7 mo vs. n.r.).

### 2.4. TCGA Cohort: All Patients

To validate these findings, we performed the same analyses in the TCGA cohort. After correlation with patient- and tumor-derived parameters, the expression of none of the genes differed significantly according to gender, tumor (T) stage (T2 vs. T3/T4), and presence of lymph node metastases. *ERCC6* was significantly more highly expressed in younger patients (<70 years, *p* = 0.0006). All other genes did not show differential expression according to patient age.

Spearman correlation analyses again revealed numerous strong positive correlations between several genes: *BRCA1* correlated with *BRCA2* (ρ = 0.6432, *p* < 0.0001), *ERCC6* (ρ = 0.1751, *p* = 0.0006), *FOXM1* (ρ = 0.6806, *p* < 0.0001), *RAD50* (ρ = 0.2389, *p* < 0.0001), and *RAD51* (ρ = 0.5626, *p* < 0.0001) and *BRCA2* correlated with *ERCC6* (ρ = 0.2911, *p* < 0.0001), *FOXM1* (ρ = 0.5119, *p* < 0.0001), *RAD50* (ρ = 0.4313, ρ < 0.0001), and *RAD51* (ρ = 0.4197, *p* < 0.0001). Furthermore, *ERCC2* correlated with *RAD52* (ρ = 0.1371, *p* = 0.0072), *ERCC6* correlated with *RAD50* (ρ = 0.3522, *p* < 0.0001), and *RAD51* (ρ = 0.1542, *p* = 0.0025) and *FOXM1* correlated with *RAD51* (ρ = 0.6294, *p* < 0.0001). Weak to moderate negative correlations were seen between *BCL2* and *RAD51*, *BRCA2* and *ERCC2*, and *ERCC2* and *ERCC6*. Detailed information is given in Table S2.

**Figure 3:**
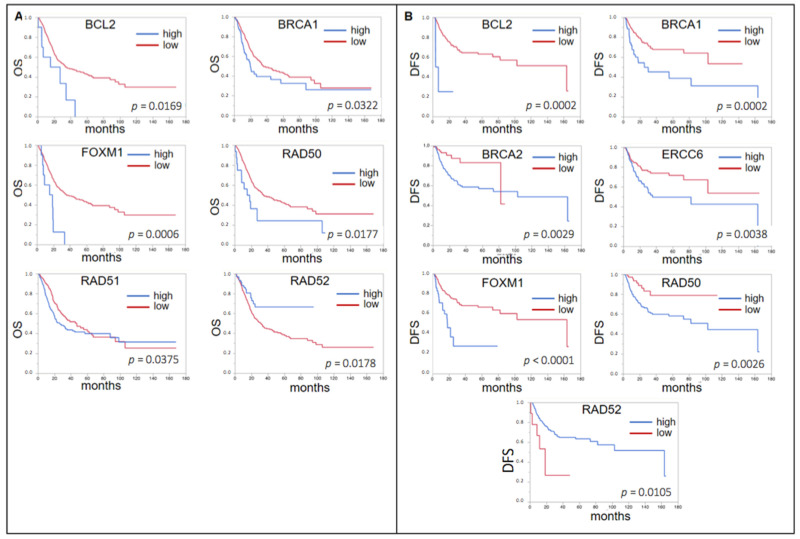
(**A**) Forrest plots of the correlation between RNA expression of DNA damage response genes (DDRG) and OS in the TCGA unselected cohort. (**B**) Forrest plots of the correlation between RNA expression of DDRG and DFS in the TCGA unselected cohort.

In the whole cohort (n = 383), only for a low expression of *RAD52* (*p* = 0.0178, median OS low 31.4 mo vs. high “not reached” (n.r.)), a significantly shorter OS was seen and *ERCC6* almost reached significance (*p* = 0.0528, 28.3 vs. 33.6 mo). Interestingly, for *BCL2* (*p* = 0.0169, 33.6 vs. 21.4 mo), *BRCA1* (*p* = 0.0322, 35.9 vs. 19.9 mo), *FOXM1* (*p* = 0.0006, 35.9 vs. 17.1 mo), *RAD50* (*p* = 0.0177, 35.5 vs. 16.7 mo), and *RAD51* (*p* = 0.0375, 47.3 vs. 26.5 mo) a high expression predicted a shorter OS (Figure 3A).

With regard to DFS, again only for *RAD52* (*p* = 0.0105, median DFS 18.6 vs. 163.7 mo), a low expression was associated with a worse prognosis, whereas the same was found for a high expression of *BCL2* (*p* = 0.0002, 163.6 vs. 5.4 mo), *BRCA1* (*p* = 0.0002, n.r. vs. 25.6 mo), *ERCC6* (*p* = 0.0038, n.r. vs. 35.5 mo), *FOXM1* (*p* < 0.0001, 163.6 vs. 18.5 mo), and *RAD50* (*p* = 0.0026, n.r. vs. 102,9 mo) (Figure 3B). *BRCA2* also showed a significant result (*p* = 0.0029) with a shorter DFS for low expression (82.4 vs. 102.9 mo). However, the curves crossed not before the last event in the low expression group.

### 2.5. TCGA Cohort: Patients Treated with ACBC

**Figure 4:**
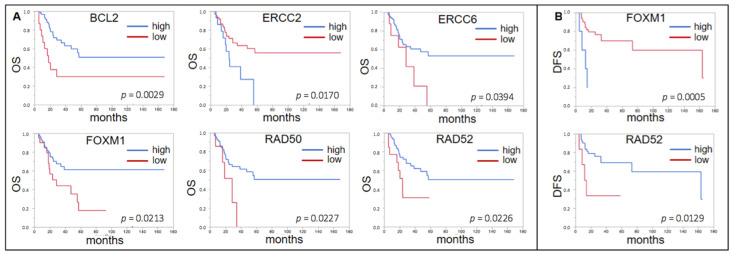
(**A**) Forrest plots of the correlation between RNA expression of DDRG and OS in the TCGA ACBC cohort. (**B**) Forrest plots of the correlation between RNA expression of DDRG and DFS in the TCGA ACBC cohort.

In those patients who received ACBC (*n* = 76) a high expression of *ERCC2* indicated a shorter OS (*p* = 0.0170, median OS n.r. vs. 24.0 mo). Similar to our own cohort, a low expression of *BCL2* (*p* = 0.0029, 18.1 vs. n.r.), *ERCC6* (*p* = 0.0394, 28.6 vs. n.r.), *FOXM1* (*p* = 0.0213, 28.8 vs. n.r.), *RAD50* (*p* = 0.0227, 28.6 vs. n.r.), and *RAD52* (*p* = 0.0226, 23.7 vs. n.r.) was indicative of a shorter OS (Figure 4A). A high expression of *FOXM1* (*p* = 0.0005, median DFS 163.6 vs. 12.6 mo) and a low expression of *RAD52* (*p* = 0.0129, 13.0 vs. 163.6 mo) indicated a shorter DFS (Figure 4B).

### 2.6. Uni- and Multivariable Analysis of the Whole TCGA Cohort Regarding Risk Factors for OS and DFS

Univariable analyses of clinical factors and DNA damage response genes in the whole cohort identified age, T stage, nodal (N) stage, *BCL2*, *BRCA1*, *FOXM1*, *RAD50*, *RAD51*, and *RAD52* as risk factors for a shorter OS. Yet, in multivariable analyses only N stage, *FOXM1*, *RAD50*, and *RAD52* remained to be independent risk factors in multivariable analyses (Table S3).

For DFS T stage, N stage, *BCL2*, *BRCA1*, *BRCA2*, *ERCC6*, *FOXM1*, *RAD50*, and *RAD52* were significant in univariable and N stage *FOXM1*, *RAD50*, and *RAD52* proved to be independent risk factors (Table S4).

### 2.7. Uni- and Multivariable Analysis of the TCGA Cohort Treated with ACBC Regarding Risk Factors for OS and DFS

In the subgroup of patients with adjuvant chemotherapy N stage, *BCL2*, *ERCC2*, *FOXM1*, and *RAD52* showed significant association with OS in univariable analyses, with N stage, *BCL2*, *ERCC2*, and *FOXM1* remaining independent (Table S5).

For DFS only N stage, *FOXM1*, *RAD50*, and *RAD52* were significant in univariable analyses. *FOXM1*, *RAD50*, and *RAD52* but not N stage remained independent risk factors (Table S6).

## 3. Discussion

In MIBC, CBC can improve outcome in responders in a neoadjuvant, as well as in an adjuvant, setting [5,15]. However, the burden of this treatment is considerable and only a fraction of patients responds and, thus, benefits.

We examined the gene expression of nine DDRG with known relevance in cisplatin resistance in a cohort of 61 patients with MIBC in an effort to identify responders. Genes were selected after literature review and in silico analysis of the TCGA set. The bcl-2 (b cell lymphoma 2) protein encoded by *BCL2* is a major apoptosis regulator and has mainly antiapoptotic effects in many different cancer entities [16]. *BRCA1* and *BRCA2* code for the correspondent breast cancer type 1/2 susceptibility proteins. They both take direct action in the repair of DNA double-strand breaks. Typically both proteins form a complex together with Palb2 (partner an localizer of *BRCA2*), which then recruits the recombinase Rad51 (encoded by *RAD51*) and the BCDX2 complex (among others consisting of several Rad51 paralogs), which is able to bind to the DNA for homologous recombinational repair [17,18]. Similar to Rad51, Rad50 (as a part of the MRN complex, encoded by *RAD50*) is also involved in the repair of DNA double-strand breaks. Yet, this complex has its main role earlier in the cascade by detecting double-strand breaks and recruiting and activating the serine-protein kinase ATM [19,20]. Rad52 (encoded by *RAD52*) is both a mediator of Rad51 function and, together with ERCC1 (DNA excision repair protein ERCC-1, encoded by *ERCC1*), is a major component in DNA repair of the single-strand annealing pathway of homologous recombination [21,22]. *ERCC6* encodes for DNA excision repair protein ERCC-6, also called CS-B protein, which has both helicase and ATPase activity, is involved both in base excision repair and nucleotide excision repair, and facilitates homologous recombination repair [23,24]. Forkhead Box M1 (encoded by FOXM1) is a transcription factor and has master regulatory effects of the transcription of multiple genes coding for proteins relevant in processes in DNA damage repair, e.g., DNA damage recognition, excision of damaged DNA, DNA unwinding, chromatin remodeling, and DNA synthesis and ligation [25].

In the unselected Mannheim cohort, irrespective of systemic treatment, *BRCA2*, *ERCC2*, *FOXM1*, and *RAD52* were associated with a shorter DFS. Regarding OS, none of the investigated genes reached statistical significance with *RAD52* showing a promising trend. Low expression correlated with poor outcome in all four genes. For *FOXM1* this is partially controversial to recent findings regarding the prognostic role of this gene in both NMIBC and MIBC [26,27].

In the TCGA set, we found that the expression of six genes correlated with OS and of seven genes correlated with DFS, in the unselected cohort. Interestingly, low expression correlated with a better outcome in six out of the seven genes, differing from our results. For *RAD52* the findings were consistent between cohorts.

Looking at the patients who received ACBC, six genes showed correlations in the Mannheim cohort (*BRCA1* and *BRCA2* for OS; *BRCA1*, *ERCC2*, *FOXM1*, *RAD50*, *RAD51*, and *RAD52 for DFS*). This is remarkable as only 20 patients were analyzed. *RAD52* had the strongest correlation, surpassing the significant correlation in the unselected cohort (*p* = 0.0059). Low expression correlated with poor outcome in all genes.

In the TCGA cohort, low expression of *BCL2*, *ERCC6*, *FOXM1*, *RAD50*, and *RAD52* correlated with decreased OS. In *RAD52* this finding was validated also in DFS. Interestingly, low expression of *FOXM1* was associated with poor OS but an improvement in DFS.

Overall, the association of low *RAD52* expression and poor outcome was consistent throughout both cohorts and also subgroups that received ACBC with DFS as an endpoint. The other way around a high expression of *RAD52* could be an indicator for a favorable response to ACBC.

Bellmunt et al. examined RNA expression of several DDRG in a cohort of 57 patients with MIBC in a similar design as the present study [28]. *ERCC1* was the only gene that could show a significant influence on OS. Low expression correlated with better outcome, differing from our cohort for this particular gene.

The reason for the partially conflicting results in several genes remains unclear. We believe that differences in the methods of analysis, next generation sequencing (NGS) for TCGA data and qRT-PCR for our own data, as well as differences in the cohort composition and ethnical and regional aspects may contribute to these differences, as we also observed this for other target genes [29].

To further investigate if this prognostic role of *RAD52* expression is independent of other risk factors we performed a multivariate analysis using logistic regression in the TCGA dataset. Here we could demonstrate that *RAD52* was highly prognostic in the whole cohort (for OS) as well as in patients treated with ACBC (for both OS and DFS).

Although there have been several studies showing the influence of mutations in DDRG on outcome and response to CBC, we chose to examine RNA expression in selected genes. Detection of mutations in DDRG requires panel sequencing and the proportion of defective genes is relatively low [11,14]. Therefore, we chose to examine the possibility to detect also functional gene alterations on the level of RNA expression, which does not necessarily need to be the result of a mutation in the respective gene.

### Limitatons

Our study has several weaknesses. The number of patients and the proportion of patients who received ACBC were limited. Furthermore, the patients were selected retrospectively. Therefore, the comparison has to be interpreted with caution. Although the effect of DDRG alterations appears to be more pronounced in patients who received ACBC, we could not differentiate between the prognostic role and the role as markers of cisplatin sensitivity. Moreover, patients who received ACBC were lymph node positive in a higher proportion, suggesting some extent of selection bias. The same applies to the TCGA data set, however.

Furthermore, our study was limited to only a subset of genes with relevance in DNA damage response. Other genes or their correspondent proteins, either directly involved in the repair of DNA damages, e.g., like phosphorylated histone family member X (γH2AX) [30] or circumvention of apoptosis, could potentially also be valuable markers for cisplatin sensitivity in MIBC.

## 4. Materials and Methods

### 4.1. Patients

After approval by the institutional ethics committee (medical faculty Mannheim, Medizinische Ethikkommission II, registration number 2015-549N-MA), a cohort of 61 patients (median age 66 years, ranging from 40–86 years, 48 males, 13 females) with MIBC who underwent radical cystectomy in the Department of Urology and Urosurgery of the University Medical Centre Mannheim was retrospectively identified (Mannheim cohort). Of these, 20 received adjuvant cisplatin-based chemotherapy (ACBC). Clinical data were extracted from medical records. All analyses were approved by the institutional review board (2016-814R-MA).

### 4.2. Selection of Genes

Candidates were selected after extensive literature review. Genes that have shown correlation to CBC response in MIBC (e.g., ERCC1, BCL2, and FOXM1) as well as genes that are associated to CBC response in other cancers were included (BRCA1, BRCA 2, RAD 51, and RAD 52). Afterwards, in silico analyses were performed with the TCGA data set. Finally, we selected nine DDRG that appeared most promising.

### 4.3. RNA Extraction and qPCR

Hematoxylin-eosin-stained (HE) 3-µm slides and subsequent 10-µm unstained slides were obtained from formalin-fixed, paraffin-embedded tissue samples. On the HE slides, areas with a high tumor content were marked and dissected from subsequent unstained slides to achieve a tumor content of at least 50% for subsequent RNA extraction, which was performed with the nucleic acid XTRACT FFPE kit (STRATIFYER Molecular Pathology GmbH, Cologne, Germany). RNA concentration and quality were measured with a Nanodrop (Thermo Fisher, Waltham, MA, USA) spectral photometer. Next, cDNA synthesis with an optimized protocol for analysis for formalin fixed paraffin embedded (FFPE) -derived samples, using a pool of sequence-specific reverse PCR primers (reference genes *RPL37A* and target genes *BCL2*, *BRCA1*, *BRCA2*, *ERCC2*, *ERCC6*, *FOXM1*, *RAD50*, *RAD51*, and *RAD52*), was performed. As reverse transcriptase, Superscript III (Thermo Fisher Scientific, Waltham, MA, USA), was used at 55 °C for 120 min, followed by an enzyme inactivation step at 70 °C for 15 min. The cDNA was stored at −20 °C or immediately used for qPCR. Forty cycles of amplification with 3 s of 95 °C and 30 s of 60 °C were conducted on a StepOnePlus qRT-PCR cycler (Applied Biosystems, Waltham, MA, USA). *RPL37A* served as housekeeping gene for normalization according to the 40-(∆Ct) method. Sequences of primers and fluorescent probes are given in Table S7. The workflow is summarized in supplementary Figure S1.

### 4.4. In Silico Analyses

The TCGA cohort for urinary bladder cancer was used for validation [31]. Clinical data and mRNA expression data were obtained from CBioPortal (https://www.cbioportal.org, accessed on 5 October 2019) [32]. Patients with non-muscle invasive bladder cancer (NMIBC) and patients with follow-up of less than three months were excluded from the subsequent analyses. Analyses with cutoff definition and Kaplan–Meier analyses were performed in analogy to the analyses in the Mannheim cohort for both the whole cohort of MIBC (*n* = 383) and those patients receiving ACBC chemotherapy (*n* = 75). Patient characteristics are given in Table 2.

### 4.5. Statistical Analyses

For statistical analyses, non-parametric two-sided *t*-test, Spearman correlation, partition test, and Kaplan–Meier analyses were used. Statistics were performed with SAS JMP 14 (SAS Institute, Cary, NC, USA) and Prism 7 (GraphPad software Inc., San Diego, CA, USA).

All methods were carried out in accordance with the relevant guidelines and regulations. Informed consent was obtained from all patients in the Mannheim cohort, prior to cystectomy.

## 5. Conclusions

RNA expression of several DDRG is correlated with DFS and OS of MIBC patients. This was also the case in the subset of patients who received ACBC, where the correlation was especially pronounced. Of the investigated genes, *RAD52* showed consistent results in both the Mannheim and the TCGA cohorts, making it a potential marker to identify patients who will likely benefit from ACBC. Although our sample size was not sufficient to draw conclusions for clinical practice, we demonstrated that RNA expression of DDRG is methodically feasible and relevant. Further investigation and validation in larger, controlled cohorts are warranted.

## Figures and Tables

**Table 1:** Patient characteristics of the Mannheim cohort.

Parameters	Whole Cohort (*n* = 61)	Patients with ACBC (*n* = 20)
Median age in years (range)	66 (40–86)	63 (40–72)
gender		
female	13	4
male	48	16
T stage ^1^ (cystectomy)		
T2	7	4
T3a	9	5
T3b	27	6
T4a	16	4
T4b	2	1
N stage ^2^		
N0	18	2
N1	21	8
N2	20	8
N3	0	2
M stage ^3^		
M0	57	20
M1	4	0
LVI ^4^		
L0	14	1
L1	39	17
n.s.	8	2
VI ^5^		
V0	32	0
V1	20	13
n.s.	9	7
Grading ^6^		
G2	9	3
G3	51	16
G4	1	1
median No of ACBC cycles	0	3
NACBC		
yes	0	0
no	61	20
Palliative chemotherapy		
yes	16	4
no	30	13
n.s. ^7^	14	3

^1^ Tumor stage; ^2^ nodal stage; ^3^ metastatic stage; ^4^ presence of lymphovascular invasion; ^5^ presence of vascular invasion; ^6^ tumor grading according to the 2016 World Health Organization (WHO) classification; ^7^ not stated.

**Table 2:** Patient characteristics of the The Cancer Genome Atlas (TCGA) cohort.

Parameters	Whole Cohort (*n* = 383)	Patients with ACBC (*n* = 75)
Median age in years (range)	69 (34–90)	66 (45–82)
gender		
female	99	20
male	284	55
T stage ^1^ (cystectomy)		
T1 (MIBC in TUR-B)	1	0
T2	113	11
T3	183	36
T4	53	17
missing (MIBC in TUR-B)	33	11
N stage ^2^		
N0	219	29
N1	46	10
N2	73	25
N3	6	2
missing	39	8
M stage ^3^		
M0	186	31
M1	11	4
MX	186	40
NACBC		
yes	10	3
no	373	72

^1^ Tumor stage; ^2^ nodal stage; ^3^ metastatic stage.

## Data Availability

Data from the TCGA cohort are available at https://www.cbioportal.org (accessed on 5 October 2019).

## Supplementary Materials

Supplementary materials can be found at https://www.mdpi.com/article/10.3390/ijms22084188/s1.

## Abbreviations

ACBC	adjuvant cisplatin-based chemotherapy
BC	bladder cancer
CBC	cisplatin-based chemotherapy
DDRG	DNA-damage response genes
DFS	disease-free survival
MIBC	muscle invasive bladder cancer
NACBC	non-muscle invasive bladder cancer
OS	overall survival
RC	radical cystectomy
TCGA	The Cancer Genome Atlas
