# Supplementary material for: RNA Expression of DNA Damage Response Genes in Muscle-Invasive Bladder Cancer: Influence on Outcome and Response to Adjuvant Cisplatin-Based Chemotherapy
Source: Int J Mol Sci. 2021 Apr 18;22(8):4188. doi: 10.3390/ijms22084188 (PMC8073847; doi:10.3390/ijms22084188)

Supplementary Materials

RNA Expression of DNA Damage Response Genes in Muscle-Invasive Bladder Cancer: Influence on Outcome and Response to Adjuvant Cisplatin-Based Chemotherapy

Table S1. Correlation of expression among the tested genes in the Mannheim cohort

gene 1	gene 2	Spearman ϱ	p value
BCL2	BRCA1	0.5575	<.0001*
BCL2	BRCA2	0.5655	<.0001*
BCL2	ERCC2	0.5972	<.0001*
BCL2	ERCC6	0.6216	<.0001*
BCL2	FOXM1	0.1621	0.2657
BCL2	RAD50	0.5595	<.0001*
BCL2	RAD51	0.4303	0.0032*
BCL2	RAD52	0.6279	<.0001*
BRCA1	BRCA2	0.8746	<.0001*
BRCA1	ERCC2	0.8252	<.0001*
BRCA1	ERCC6	0.6919	<.0001*
BRCA1	FOXM1	0.7054	<.0001*
BRCA1	RAD50	0.8008	<.0001*
BRCA1	RAD51	0.8525	<.0001*
BRCA1	RAD52	0.7044	<.0001*
BRCA2	ERCC2	0.7823	<.0001*
BRCA2	ERCC6	0.7086	<.0001*
BRCA2	FOXM1	0.7152	<.0001*
BRCA2	RAD50	0.789	<.0001*
BRCA2	RAD51	0.8237	<.0001*
BRCA2	RAD52	0.7323	<.0001*
ERCC2	ERCC6	0.7669	<.0001*
ERCC2	FOXM1	0.6945	<.0001*
ERCC2	RAD50	0.7561	<.0001*
ERCC2	RAD51	0.7943	<.0001*
ERCC2	RAD52	0.7618	<.0001*
ERCC6	FOXM1	0.5481	<.0001*
ERCC6	RAD50	0.8286	<.0001*
ERCC6	RAD51	0.625	<.0001*
ERCC6	RAD52	0.7608	<.0001*
FOXM1	RAD50	0.586	<.0001*
FOXM1	RAD51	0.7994	<.0001*
FOXM1	RAD52	0.6222	<.0001*
RAD50	RAD51	0.7596	<.0001*
RAD50	RAD52	0.8237	<.0001*
RAD51	RAD52	0.7393	<.0001*

Table S2. Correlation of expression among the tested genes in the TCGA cohort

gene 1	gene 2	Spearman ρ	p value
BCL2	BRCA1	0.0423	0.4087
BCL2	BRCA2	0.047	0.3593
BCL2	ERCC2	0.0339	0.5077
BCL2	ERCC6	-0.0971	0.0577
BCL2	FOXM1	-0.0136	0.7911
BCL2	RAD50	0.004	0.9382
BCL2	RAD51	-0.1403	0.0060*
BCL2	RAD52	0.0038	0.9414
BRCA1	BRCA2	0.6432	<.0001*
BRCA1	ERCC2	-0.0480	0.3486
BRCA1	ERCC6	0.1751	0.0006*
BRCA1	FOXM1	0.6806	<.0001*
BRCA1	RAD50	0.2389	<.0001*
BRCA1	RAD51	0.5626	<.0001*
BRCA1	RAD52	-0.0147	0.7738
BRCA2	ERCC2	-0.1395	0.0062*
BRCA2	ERCC6	0.2911	<.0001*
BRCA2	FOXM1	0.5119	<.0001*
BRCA2	RAD50	0.4313	<.0001*
BRCA2	RAD51	0.4197	<.0001*
BRCA2	RAD52	0.054	0.2916
ERCC2	ERCC6	-0.1071	0.0362*
ERCC2	FOXM1	-0.0445	0.3855
ERCC2	RAD50	-0.2261	<.0001*
ERCC2	RAD51	-0.0492	0.3373
ERCC2	RAD52	0.1371	0.0072*
ERCC6	FOXM1	-0.0015	0.9768
ERCC6	RAD50	0.3522	<.0001*
ERCC6	RAD51	-0.0355	0.4888
ERCC6	RAD52	0.1542	0.0025*
FOXM1	RAD50	0.0174	0.7343
FOXM1	RAD51	0.6294	<.0001*
FOXM1	RAD52	-0.0837	0.1019
RAD50	RAD51	0.0046	0.9278
RAD50	RAD52	0.0474	0.3549
RAD51	RAD52	-0.0939	0.0663

Table S3. Uni- and multivariable analysis of the whole TCGA cohort regarding risk factors for OS

	univariable analysis		multivariable analysis	
**Parameter**	**p value**	**HR (95% CI)**	**p value**	**HR (95% CI)**
Gender (male vs. female)	0.5429	0.9025 (0.6548-1.2634)	**-**	**-**
Age (≥ 70 vs. <70 years)	**0.0036**	1.5529 (1.1548-2.0939)	**0.0065**	1.5680 (1.1342-2.1746)
T stage (T3/4 vs. T2)	**<0.0001**	1.9312 (1.3894-2.7325)	**0.0079**	1.6832 (1.1420-2.5428)
N stage (positive vs. negative)	**<0.0001**	2.2729 (1.6581-3.1197)	**<0.0001**	2.1397 (1.5270-3.0087)
BCL2 (high vs. low)	**0.0398**	2.3195 (1.0450-4.4162)	0.1032	2.0471 (0.8495-4.1861)
BRCA1 (high vs. low)	**0.0405**	1.4780 (1.0176-2.0948)	0.6181	1.1235 (0.7026-1.7478)
BRCA2 (high vs. low)	0.3207	1.1967 (0.8439-1.7386)	-	-
ERCC2 (high vs. low)	0.3590	1.5411 (0.6528-5.0059)	-	-
ERCC6 (high vs. low)	0.0710	0.6272 (0.4000-1.0435)	-	-
FOXM1 (high vs. low)	**0.0053**	3.0670 (1.4479-5.6826)	**0.0097**	3.0962 (1.3547-6.1585)
RAD50 (high vs. low)	**0.0348**	2.0119 (1.0560-3.4726)	**0.0358**	2.1767 (1.0564-4.1681)
RAD51 (high vs. low)	**0.0367**	1.3742 (1.0196-1.8622)	0.5088	1.1211 (0.7988-1.5778)
RAD52 (high vs. low)	**0.0112**	0.5419 (0.3113-0.8774)	**0.0255**	0.5486 (0.2949-0.9335)

Table S4. Uni- and multivariable analysis of the whole TCGA cohort regarding risk factors for DFS

		**univariable analysis**		**multivariable analysis**
**Parameter**	**p value**	**HR (95% CI)**	**p value**	**HR (95% CI)**
Gender (male vs. female)	0.9489	0.9839 (0.6117-1.6493)	-	-
Age (≥ 70 vs. <70 years)	0.8801	1.0336 (0.6691-1.5839)	-	-
T stage (T3/4 vs. T2)	**0.0200**	1.6973 (1.0852-2.722)	0.0653	1.7028 (0.9679-3.1487)
N stage (positive vs. negative)	**<0.0001**	2.6801 (1.6915-4.2380)	**0.0044**	2.0102 (1.2455-3.2431)
BCL2 (high vs. low)	**0.0127**	6,7182 (1.6405-18.1082)	0.1567	1.4802 (0.8647-2.6589)
BRCA1 (high vs. low)	**0.0006**	2.4018 (1.476-3,7915)	0.1958	1.4667 (0.8152-2.5564)
BRCA2 (high vs. low)	**0.0012**	2.7397 (1.4490-5.8781)	0.1591	1.6784 (0.8275-3.8764)
ERCC2 (high vs. low)	0.2330	0.7633 (0.4809-1.1861)	-	-
ERCC6 (high vs. low)	**0.0043**	1.8552 (1.2139-2.8514)	0.0689	1.5617 (0.9661-2.5425)
FOXM1 (high vs. low)	**0.0003**	2.9688 (1.7023-4.9233)	**0.0274**	2.0122 (1.0848-3.5729)
RAD50 (high vs. low)	**0.0010**	2.7642 (1.4631-5.9277)	**0.0270**	2.2583 (1.0893-5.4851)
RAD51 (high vs. low)	0.2284	0.7547 (0.4652-1.1877)	-	-
RAD52 (high vs. low)	**0.0374**	0.3250 (0.1452-0.9269)	**0.0119**	0.1881 (0.0710-0.6513)

Table S5. Uni- and multivariable analysis of patients from the TCGA cohort treated with adjuvant chemotherapy regarding risk factors for OS

	univariable analysis		multivariable analysis	
**Parameter**	**p value**	**HR (95% CI)**	**p value**	**HR (95% CI)**
Gender (male vs. female)	0.2944	1.6256 (0.6786-4.8087)	-	-
Age (≥ 70 vs. <70 years)	0.6802	0.8389 (0.3323-1.8607)	-	-
T stage (T3/4 vs. T2)	0.8726	1.0759 (0.4658-2.9181)	-	-
N stage (positive vs. negative)	**0.0006**	4.910 (1.8788-16.8049)	**0.0080**	3.7718 (1.3804-13.2667)
BCL2 (high vs. low)	**0.0087**	0.3336 (0.1601-0.7433)	**0.0133**	0.2567 (0.0896-0.7457)
BRCA1 (high vs. low)	0.1638	2.2884 (0.6748-5.8778)	-	-
BRCA2 (high vs. low)	0.1732	1.8640 (0.7777-5.5162)	-	-
ERCC2 (high vs. low)	**0.0296**	2.4633 (1.0997-5.1818)	**0.0051**	4.0301 (1.5447-10.5204)
ERCC6 (high vs. low)	0.0702	0.4020 (0.1747-1.0876)	-	-
FOXM1 (high vs. low)	**0.0304**	0.4420 (0.2176-0.9228)	**0.0241**	0.3673 (0.1509-0.8755)
RAD50 (high vs. low)	0.0532	0.3392 (0.1382-1.0169)	**-**	-
RAD51 (high vs. low)	0.1573	0.4032 (0.0649-1.3459)	-	-
RAD52 (high vs. low)	**0.0415**	0.3998 (0.1839-0.9624)	0.0919	0.4085 (0.1608-1.1727)

Table S6. Uni- and multivariable analysis of patients from the TCGA cohort treated with adjuvant chemotherapy regarding risk factors for DFS

	univariable analysis		multivariable analysis	
**Parameter**	**p value**	**HR (95% CI)**	**p value**	**HR (95% CI)**
Gender (male vs. female)	0.2612	1.9411 (0.6403-8.4032)	-	-
Age (≥ 70 vs. <70 years)	0.8776	0.9221 (0.2944-2.4629)	-	-
T stage (T3/4 vs. T2)	0.7271	0.8277 (0.3055-2.6106)	-	-
N stage (positive vs. negative)	**0.0344**	2.7845 (1.0769-8.0268)	0.0596	2.5405 (0.9636-7.4165)
BCL2 (high vs. low)	0.1375	0.4218 (0.1553-1.3584)	-	-
BRCA1 (high vs. low)	0.5656	1.3284 (0.5144-3.8239)	-	-
BRCA2 (high vs. low)	0.0543	2.5904 (0.9832-8.0611)	-	-
ERCC2 (high vs. low)	0.1019	2.8220 (0.7905-8.0236)	-	-
ERCC6 (high vs. low)	0.1133	2.8216 (0.7550-8.1934)	-	-
FOXM1 (high vs. low)	**0.0092**	6.1705 (1.6682-19.0089)	**0.0025**	9.8701 (2.4558-35.9704)
RAD50 (high vs. low)	**0.0137**	0 (0.6108-0.6108)	**0.0323**	0 (0-0.8216)
RAD51 (high vs. low)	0.2175	0.3306 (0.0179-1.6901)	-	-
RAD52 (high vs. low)	**0.0417**	0.2644 (0.0927-0.9440)	**0.0128**	0.1612 (0.0454-0.6501)

Table S7. Primers and probes that were used in this study

Gene	Forward Primer Sequence (5`→ 3`)	Reverse Primer Sequence (5`→ 3`)	Probe Sequence (5`label → 3`label)
BCL2	GTGGACAACATCGCCCTG	CACAAAGGCATCCCAGCC	FAM-GGATGACTGAGTACCTG AACCGGCACCTG-BHQ1
BRCA1	CTTAGAGTGTCCCATCTGT CTGG	GCCCTTTCTTCTGGTTGAG A	FAM-GTGGTCACACTTTGTGG AGACAGGTTCCTTGATC-BHQ1
BRCA2	GAGATAAGTCAGTGGTATG TGGG	GTGGTGTAGCTAAAGAACT TGACC	FAM-GTCTAGGAGCTGAGGT GGATCCTGATATGT-BHQ1
ERCC2	AGCTCAAACGCACGCTGG	GCGGATATGCTCTCTGGTA TGC	FAM-CGCCAAGGGTCATGGA GTCCTGGAGATGC-BHQ1
ERCC6	AGGAGGATGCAGAGCCGG	ACCAAAAGGTGTCATCTGG CC	FAM-CCATCCAGTCTTGGCAG CATGCTCATGCCT-BHQ1
FOXM1	GACCACCTGGAGCCCTTT G	GATGTTGGATAGGCTATTG TTGATAGTG	FAM-CAGAAACGGGAGACC TGTGATGGTGAGGCAG-BHQ1
RAD50	TTGAACAGGGTCGTCTACA GC	CATCCAATTCTAGCTGTGT TGCC	FAM-CTCGGATATGTTCTTGA TGGCGATCTGCTTGC-BHQ1
RAD51	CACGGTTAGAGCAGTGTG GC	CTCCTTCTTTGGCGCATAG GC	FAM-CAGCCTCCACAGTATGG AATCCAGCTTCTTCC-BHQ1
RAD52	GGTTATGGTGTTAGTGAGG GCC	CCAAAACTCCTGAGGGCTC G	FAM-GGAAGGAGGCGGTGAC AGACGGGCTG-BHQ1
RPL37A	TGTGGTTCCTGCATGAAGA CA	GTGACAGCGGAAGTGGTA TTGTAC	FAM-TGGCTGGCGGTGCCTGGA-BHQ1

Figure S1. Workflow of experiments

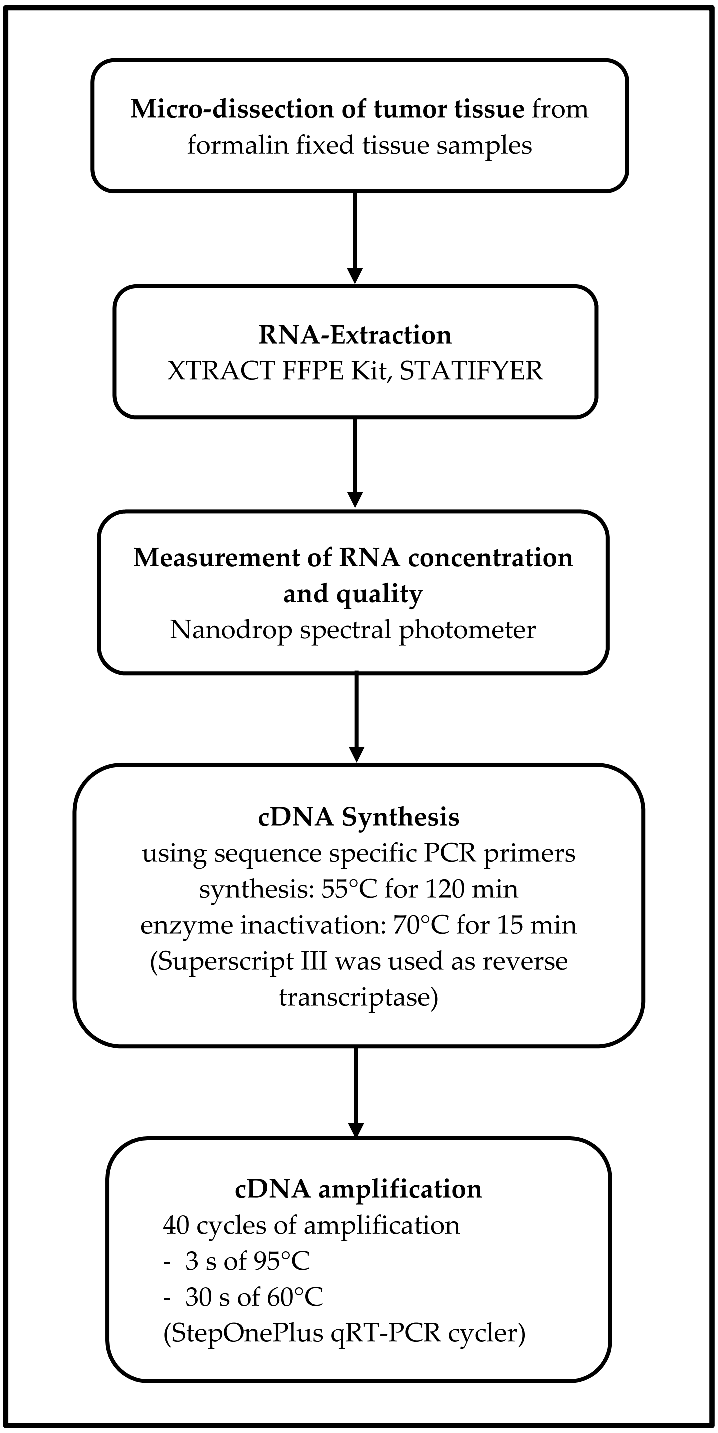

Supplement: Supplementary file 1 [file ijms-22-04188-s001.zip › ijms-1169491-supplementary.pdf]
